# Pulmonary talc granulomatosis mimicking malignant disease 30 years after last exposure: a case report

**DOI:** 10.1186/1752-1947-2-225

**Published:** 2008-07-03

**Authors:** William S Krimsky, Suneel Dhand

**Affiliations:** 1 Johns Hopkins University School of Medicine, Baltimore, MD, USA; 2 Franklin Square Hospital Center, Baltimore, MD, USA; 3 The Delmarva Foundation for Medical Care, Cambridge, MA, USA

## Abstract

**Introduction:**

Pulmonary talc granulomatosis is a rare disorder characterized by the development of foreign body granuloma secondary to talc exposure. Previous case reports have documented the illness in current intravenous drug users who inject medications intended for oral use. We present a rare case of the disease in a patient with a distant history of heroin abuse who presented initially with history and imaging findings highly suggestive of malignancy.

**Case presentation:**

A 53-year-old man reported a 4-month history of increasing dyspnea and weight loss. He had a long history of smoking and admission chest X-ray revealed a density in the right hemithorax. Computed tomography confirmed a probable mass with further speculated opacities in both lung fields suspicious for malignant spread. Biopsies obtained using endobronchial ultrasound-guided aspiration returned negative for malignancy and showed bronchial epithelial cells with foreign body giant cell reaction and polarizable birefringent talc crystals.

**Conclusion:**

This case demonstrates a rare presentation of talc granulomatosis three decades after the last likely exposure. The history and imaging findings in a chronic smoker were initially strongly suggestive of malignant disease, and we recommend that talc-induced lung disease is considered in any patient with multiple scattered pulmonary lesions and a history of intravenous drug use. Confirmation of the disease by biopsy is essential, but unfortunately there are few successful proven management options for patients with worsening disease.

## Introduction

Pulmonary talc granulomatosis is a rare disorder characterized by the development of foreign body granuloma secondary to talc exposure. Several case reports have documented the disease in known intravenous drug abusers who present with respiratory symptoms. We present the diagnosis in a patient with a remote history of intravenous heroin use, and initial symptoms and imaging suggestive of malignancy.

## Case presentation

A 53-year-old man presented with increasing dyspnea and a weight loss of 3 kg over a 4-month period. Past medical history was significant for emphysema, seizure disorder and hepatitis C. Medications included albuterol and dilantin. The patient was unemployed and had a 35-pack/year history of smoking. He also reported intravenous heroin abuse 30 years previously (undertaken for a period of 10 years).

**Figure 1:**
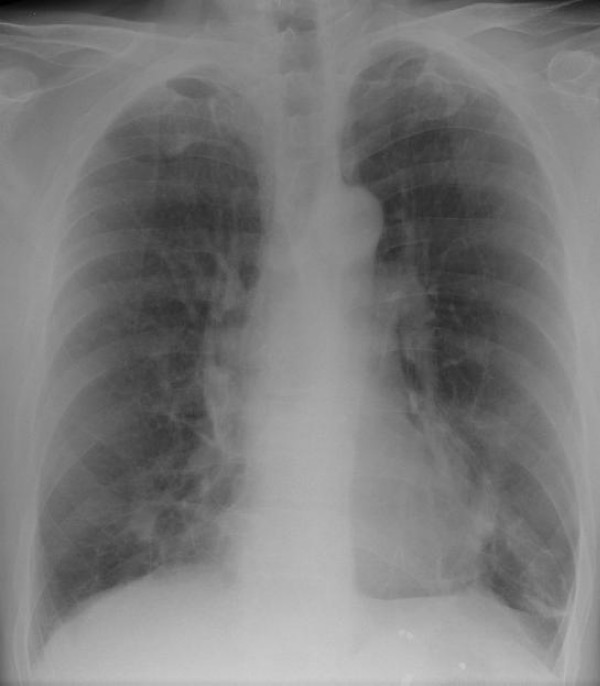
Ill-defined, mass-like density in the right middle lobe of the lung.

**Figure 2:**
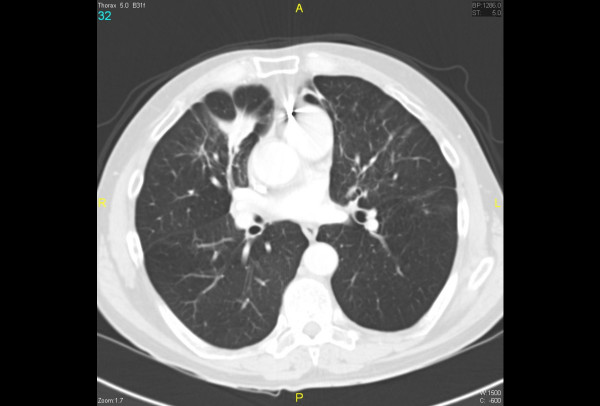
**Spiculated opacity suspicious for lung malignancy**. Maximum diameter 4.5 cm.

Laboratory results including complete blood count, renal function and liver function tests were all within normal limits. Human immunodeficiency virus (HIV) serology was negative. A chest X-ray (Figure 1) showed an ill-defined density close to the right heart border, and a computed tomography (CT) scan (Figure 2) confirmed a 4.5 × 2.2 cm opacity in the medial aspect of the right middle lobe, with emphysematous changes and spiculated opacities in both lung fields suspicious for malignant spread. A CT scan of the abdomen and pelvis was unremarkable.

The patient underwent autofluorescence bronchoscopy and the visualized portions of the upper and lower airways were widely patent with no abnormalities. Ultrasound of the mediastinum using ultrasonic bronchofibervideoscope located the echodensity inferior to the right hilum, and fine needle biopsy of this structure was obtained via endobronchial ultrasound-guided transbronchial needle aspiration. Biopsies were also obtained from four further echodense areas suggestive of malignant lesions involving both lungs.

**Figure 3:**
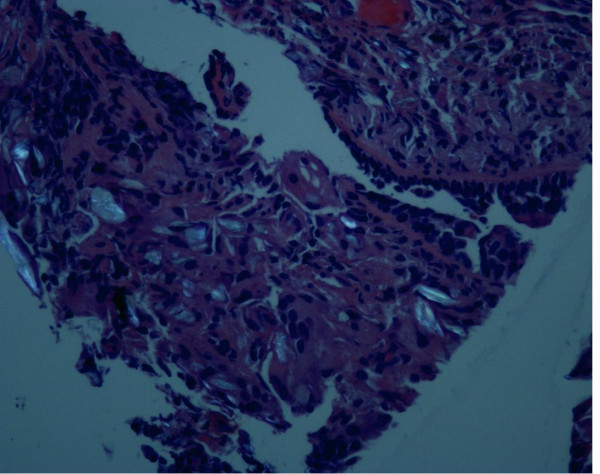
**Histology showing talc particles**. Hematoxylin-eosin stain, magnification ×400.

**Figure 4:**
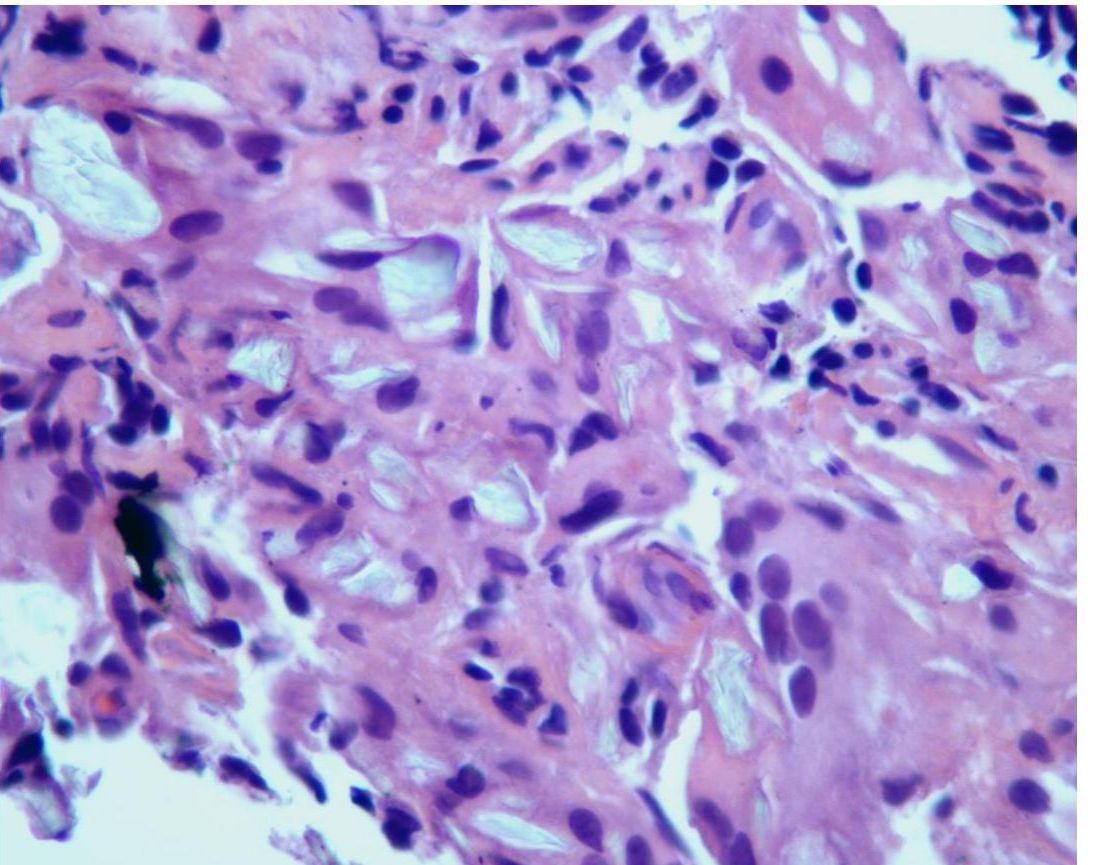
**Talc particles under polarized light**. Magnification ×200.

**Figure 5:**
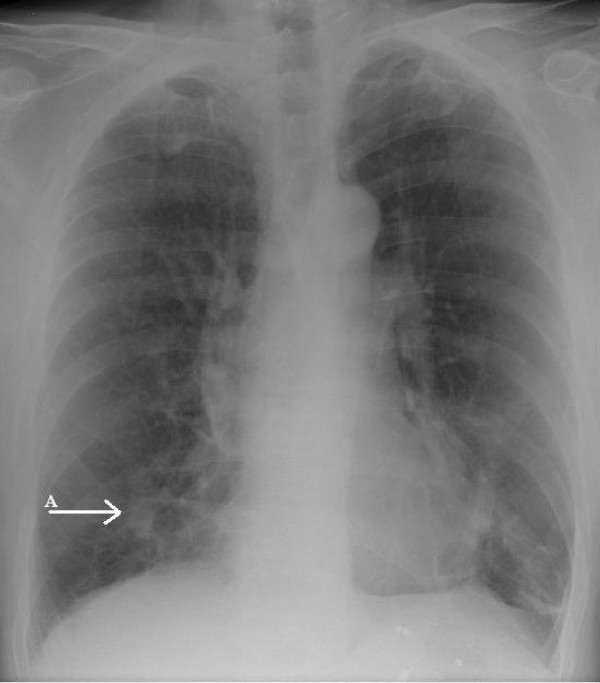
**Histology showing talc particles at a lower magnification**. Hematoxylin-eosin stain.

The biopsy returned negative for malignancy, and histology from the multiple sites showed bronchial epithelial cells with a marked foreign body giant cell reaction and associated polarizable birefringent foreign bodies (Figures 3, 4 and 5). A diagnosis of talc granulomatosis secondary to previous intravenous drug abuse was made. The patient was discharged home and his dyspnea and weight loss were attributed to worsening emphysema in the setting of continued heavy smoking, superimposed on talc granulomatosis, causing deteriorating lung infection.

## Discussion

Talc, or hydrated magnesium silicate, is formed during the breakdown of anthrophyllite rock. It has a wide array of industrial uses, and for pharmacological purposes is utilized as a binder in oral tablets to hold the medication together.

Four types of pulmonary disease secondary to talc exposure have been defined [1]: (i) talcosilicosis, (ii) talco-asbestosis, (iii) talcosis and (iv) talc granulomatosis. The first two are associated with occupational exposure, the third with inhalation of pure talc, and the fourth in the setting of intravenous drug addicts who inject tablets intended for oral use.

It is probable that only a small percentage of intravenous drug addicts frequently engage in this activity [2], and medications associated with the disease include methylphenidate, methadone and amphetamines. In addition to drug abusers who inject these oral medications, talcosis has also been reported secondary to cocaine sniffing in the absence of any intravenous drug use [3].

The exact pathophysiological mechanism of talc granulomatosis is unknown. Talc embolization results in an initial inflammatory arteritis, associated with a rapid influx of neutrophils around the talc particle. Foreign body granuloma later develops after migration of particles to the surrounding perivascular and pulmonary interstitial tissue.

This reaction to talc is highly variable, some patients develop interstitial granulomas and others develop granulomas in the lumens of small pulmonary arteries (resulting in pulmonary hypertension). The carcinogenicity of inhaled talc also remains controversial, with one study finding no increase in the rate of lung cancer in employees of a talc processing plant in New York over a 31-year period [4].

Patients with talc granulomatosis can range from asymptomatic to fulminant disease. Symptomatic patients typically present with non-specific complaints including progressive exertional dyspnea, dry cough, or less typically, weight loss and night sweats. More serious presentations may involve adult respiratory distress syndrome or progressive massive fibrosis [5]. Spontaneous pneumothorax has also been reported as a presenting symptom [6]. Further cases of extrapulmonary disease have been documented in the eyes (talc retinopathy) [7], uterus [8] and liver [9].

Physical examination is typically unremarkable, although there may be bibasal crepitations in the presence of fibrosis. Laboratory values are also usually within normal limits. Pulmonary function tests frequently show a reduction in carbon monoxide diffusion.

The most common chest X-ray finding is widespread, 2 to 3 mm, well-defined nodules, often in the mid-lung [10]. As the disease progresses, the nodules coalesce and massive fibrosis can occur. High-resolution CT may reveal a diffuse ground-glass pattern with emphysema. One review of CT findings in 12 patients with talc granulomatosis found that the predominant abnormalities were nodules and lower lobe panacinar emphysema (three patients), diffuse fine nodular pattern (two patients), and ground-glass attenuation (two patients). Emphysema was seen in the remaining five patients [11].

The patient in our case had a relatively good baseline level of health and had been free of drug use for 30 years. He had no other hospitalizations or outpatient investigations that had previously demonstrated the presence of any abnormalities in his chest.

Differential diagnoses include: interstitial lung disease, emphysema, sarcoidosis, pneumoconiosis, tuberculosis and opportunistic infections such as pneumocystis and cytomegalovirus pneumonia. Neoplasms, such as broncho-alveolar carcinoma and lymphoid malignancy, must also be excluded. Any patient with risk factors should be tested for HIV.

Bronchoscopy and biopsy are necessary for definitive diagnosis. Early diagnosis is paramount in order to avoid misdiagnosis. A single case report of a 38-year-old man with HIV reported empirical treatment for *Pneumocystis jiroveci *in a patient who subsequently died before the correct diagnosis was made [12].

Broncho-alveolar lavage will usually reveal lymphocytosis with a predominance of CD8 lymphocytes. The fluid appears crystalline under polarized light. Transbronchial or open-lung biopsy can be utilized to obtain a tissue specimen. Histology will reveal foreign body giant cell reaction with birefringent plate-like talc crystals.

There are no established treatments for talc granulomatosis. Patients must stop intravenous drug and any tobacco use. Successful steroid use has been reported in a 24-year-old man who responded to treatment with 60 mg of prednisone daily [13], but further data are lacking. Associated pulmonary hypertension should be treated with vasodilators. Lung transplantation is reserved as a last resort for patients with end-stage disease. Unfortunately most patients have poor outcomes and experience a progressive decline in pulmonary function. One 10-year follow-up of six patients described an irreversible progression of radiographic abnormalities [5].

## Conclusion

This case demonstrates a rare presentation of talc granulomatosis three decades after the last likely exposure. The history and imaging findings in a chronic smoker were initially strongly suggestive of malignant disease, and we recommend that talc-induced lung disease is considered in any patient with multiple scattered pulmonary lesions and a history of intravenous drug use. Confirmation of the disease by biopsy is essential, but unfortunately there are few successful proven management options for patients with worsening disease.

## Competing interests

The authors declare that they have no competing interests.

## Consent

Written informed consent was obtained from the patient for publication of this case report and accompanying images. A copy of the written consent is available for review by the Editor-in-Chief of this journal.

## Authors' contributions

SD carried out the background research and put the manuscript together. WM performed the bronchoscopy and was the Senior Attending physician. WM supervised and gave ideas on the manuscript preparation. Both SD and WM conceived the initial idea. All authors read and approved the final manuscript.
